# Appendix to Article V. P. 81, on the Medical Jurisprudence of Insanity

**Published:** 1843-07

**Authors:** 


### Appendix *to the Article on the Medical Jurisprudence of Insanity.*

(Art. V. p. 81.)

We are desirous of communicating to our readers in the present Number the answers returned by the fifteen judges to the questions on the "Plea of Insanity," submitted to them by the House of Lords. It will be obvious that several of these questions arose directly out of the trial of M'Naughten for the murder of Mr. Drummond. The answers were read in the name of all the judges, excepting one, (Mr. Justice Maule,) by Lord Chief Justice Tindal, on the 19th June, 1843.

Question I. What is the law respecting alleged crimes committed by persons afflicted with insane delusion, in respect of one or more particular subjects or persons: as for instance, where at the time of the commission of the alleged crime the accused knew he was acting contrary to law, but did the act complained of with the view, under the influence of some insane delusion, of redressing or avenging some supposed grievance or injury, or of producing some supposed public benefit?

Answer. The opinion of the judges was that notwithstanding the party committed a wrong act, while labouring under the idea that he was redressing a supposed grievance or injury, or under the impression of obtaining some public or private benefit, he was liable to punishment.

Quest. II. What are the proper questions to be submitted to the jury, when a person alleged to be afflicted with insane delusion, respecting one or more particular subjects or persons, is charged with the commission of a crime, murder for example, and insanity is set up as a defence?

Ans. The jury ought in all cases to be told that every man should be considered of sane mind until the contrary were clearly proved in evidence. That before a plea of insanity should be allowed, undoubted evidence ought to be adduced that the accused was of diseased mind, and that at the time he committed the act he was not conscious of right or wrong. This opinion related to every case in which a party was charged with an illegal act, and a plea of insanity was set up. Every person was supposed to know what the law was, and therefore nothing could justify a wrong act except it was clearly proved that the party did not know right from wrong. If that was not satisfactorily proved the accused was liable to punishment; and it was the duty of the judge so to tell the jury when summing up the evidence, accompanied by those remarks and observations which the nature and peculiarities of each case might suggest and require.

Quest. III. In what terms ought the question to be left to the jury as to the prisoner's state of mind at the time when the act was committed?

No answer was returned to this question.

Quest. IV. If a person, under an insane delusion as to existing facts, commits an offence in consequence thereof, is he thereby excused?

Ans. If the delusion were only partial, the party accused was equally liable with a person of sane mind. If the accused killed another in self-defence he would be entitled to an acquittal, but if the crime were committed for any supposed injury he would then be liable to the punishment awarded by the laws to his crime.

Quest. V. Can a medical man, conversant with the disease of insanity, who never saw the prisoner previously to the trial, but who was present during the whole trial and the examination of all the witnesses, be asked his opinion as to the state of the prisoner's mind at the time of the commission of the alleged crime, or his opinion whether the prisoner was conscious, at the time of doing the act, that he was acting contrary to law? or whether he was labouring under any, and what delusion at the time?

Ans. The question could not be put in the precise form stated above, for by doing so it would be assumed that the facts had been proved. When the facts were proved and admitted, then the question, as one of science, would be generally put to a witness under the circumstances stated in the interrogatory.

Mr. Justice Maule agreed with the judges in respect to the answers returned to all the questions excepting the last; from this he entirely dissented. In his opinion, such questions might be at once put to medical men without reference to the facts proved; and he considered that this had been done, and the legality of the practice thereby confirmed on the trial of M'Naughten.

The present state of the law of England, relative to the plea of insanity in criminal cases may be considered to be embodied in the foregoing replies. To us it appears that the question is left much in the state in which, before these queries were put, it was known to exist. These queries embrace all the difficulties suggested in the article on the Medical Jurisprudence of Insanity; we wish we could say that the replies had removed them. One point has been satisfactorily elicited, namely, that there is great unanimity in the views entertained on this subject by our high law authorities; and in future therefore we anticipate there will be no reason to complain of conflicting decisions in the trial of these cases.

From the reply to the first question we learn that the existence of delusion in the commission of a crime will not excuse it, if the party knew he was acting contrary to law. It is, we think, obvious that this answer admits of a very wide construction; for how is it to be determined, that a man committing an act while labouring under a delusion knew that he was acting contrary to law? Applying the principle here set forth to the case of Martin, the incendiary of York cathedral, it is pretty certain that he was liable to punishment, and yet he was acquitted. Applying it to Mr. M'Naughten's case, we must infer that the accused did not know that in shooting Mr Drummond he was acting contrary to law,—the very point on which so much dissatisfaction with respect to the verdict arose. Hence in all similar cases a like inference may be drawn.

The reply to the second question brings before us the old test of insanity in the perpetration of crime, namely, the consciousness of right or wrong in the mind of the perpetrator at the time of its commission. We have elsewhere remarked upon the insufficiency of this test; and, therefore, it does not require to be further considered in this place. The reply to the fourth question shows that partial delusion does not excuse a person who has committed a crime, except in those cases where a sane man would be equally excused. It would appear further, that if murder were committed for an imaginary injury, the fact of the person labouring under partial delusion as to existing facts would not excuse the crime. To apply this to M'Naughten's case, we find that there was here "a supposed injury," as he considered the deceased to be one of his persecutors, Therefore, in order to reconcile the verdict with this legal doctrine, we must infer that M'Naughten's delusion was not partial but general.

We have elsewhere expressed our opinion as to the form in which questions should be put to medical witnesses; and that view is in the reply to the fifth question, borne out by the opinions of fourteen judges out of fifteen. It is now decided that facts tending to lead to a strong suspicion of insanity must be proved and admitted before the opinions of medical witnesses can be received.

